# The National Cat-Show

**Published:** 1895-05

**Authors:** 


THE National Cat Show to be held in New York City from May 8th to 11th inclusive, will prove an interesting and attractive one to all lovers of the feline species. The long experience in other animal shows of the managers will insure thorough care and fairness. Some fifty-four prizes, in as many varieties and classes, will be offered for competition.

